# Prevalence, Treatment, and Associated Factors of Hypertension in Spain: A Comparative Study between Populations

**DOI:** 10.1155/2018/4851512

**Published:** 2018-08-14

**Authors:** Arturo Corbatón-Anchuelo, María Teresa Martínez-Larrad, Náyade del Prado-González, Cristina Fernández-Pérez, Rafael Gabriel, Manuel Serrano-Ríos

**Affiliations:** ^1^Instituto de Investigación Sanitaria, Hospital Clínico San Carlos (IdISSC), Madrid, Spain; ^2^Spanish Biomedical Research Centre in Diabetes and Associated Metabolic Disorders (CIBERDEM), Madrid, Spain; ^3^Escuela Nacional de Salud, ISCIII, Spain

## Abstract

The prevalence and related factors of hypertensive subjects according to the resident area (rural versus urban) were investigated in two population-based studies from Spain. Medical questionnaires were administered and anthropometrics were measured, using standardized protocols. Hypertension was diagnosed in pharmacology treated subjects or those with blood pressure (BP) ≥140/90 mm Hg. Regarding BP control, it was defined as under control if BP was <140/90 or <140/85 mm Hg in type 2 diabetic subjects. Information on educational status, social class, smoking habit, and alcohol intake was obtained. 3,816 subjects (54.38 % women) were included. Prevalence of diagnosed hypertension was higher in women and showed no differences according to the living area (men: urban 21.88 versus rural 21.92 %, p = 0.986; women: urban 28.73 versus rural 30.01 %, p = 0.540). Women living in rural areas and men with secondary or tertiary education levels had a lower probability of being BP uncontrolled (OR (95 % CI): 0.501 (0.258–0.970)/p=0.040, 0.245 (0.092–0.654)/p=0.005, and 0.156 (0.044–0.549)/p=0.004, respectively). Urban young men (31-45 years) and medium aged women (46-60 years) were less BP controlled than their rural counterparts (41.30 versus 65.79 %/p=0.025 and 35.24 versus 53.27 %/p=0.002, respectively).

## 1. Introduction

Hypertension is one of the most important risk factors for cardiovascular, cerebrovascular, and peripheral vascular diseases as well as end-stage renal disease, together with diabetes mellitus, dyslipidemia, and smoking. These factors are significant contributors to deaths and disability in the developed countries [1]. In a recently published study in Catalonia (Spain), hypertension and lipid disorders were the most prevalent founded pair of chronic disorders in subjects older than 45 years old [2]. Regional differences and a gradient from northwest to southeast in adiposity and cardiovascular morbidity and mortality have been widely demonstrated in previous studies in Spain [3], but oppositely to other ethnic populations and countries [4–6], we lack studies on cardiovascular risk factors, specifically hypertension, comparing rural and urban areas. In fact, some recently described strategies on healthy lifestyle have shown to lower blood pressure (BP), reducing the risk of complications associated with hypertension [7–9]. Differences on diet and physical activities have also been found and described in rural and urban areas across Spain [10–12], and, therefore, we believe that there are differences in the prevalence and characteristics of hypertension as well as in the associated factors.

The* Spanish Insulin Resistance (SIRS) *and the* Segovia Insulin Resistance *population-based studies were carried out by well-trained personnel in rural and urban areas of six autonomous communities in Spain, with the aim of knowing the prevalence of Metabolic Syndrome (MetS) and its associated cardiovascular risk factors. The prevalence of glucose tolerance categories and MetS was recently reported [13]. In conclusion, we found that MetS prevalence according to the most recent Harmonized Criteria remained stable in the last decade in Spanish females but slightly increased in males, with about one out of three men affected. Moreover, one out of four subjects had prediabetes. Thus, in this study our aim was to describe the prevalence and characteristics of hypertensive subjects as well as blood pressure control, according to the resident area.

## 2. Materials and Methods

We studied two Spanish cohorts focused on cardiovascular risk factors, whose recruitment procedures have been previously reported [13]: (A) The* Spanish Insulin Resistance Study (SIRS) *[14] is a cross-sectional population-based study carried out in 7 small and middle-size towns across Spain. It was estimated that it would be necessary to recruit a random sample of 3,000 individuals from a targeted population of 348,980 inhabitants aged 35 to 69 years old to get a precision lower than 2 % for a 20 % MetS prevalence [15]. To get this appropriate sample size, we selected 5,363 subjects from the census with the following result: 1,177 (21.9 %) census errors, 1,014 (18.9 %) refused, 3,172 accepted (response rate, 75.8 %), 147 met exclusion criteria, and 92 did not complete the study for diverse reasons. Finally, 4 subjects missed some clinical data, so 2,929 men and nonpregnant women were included in the current analysis. (B) The* Segovia Insulin Resistance Study* [12, 16], cross-sectional population-based study in the Spanish province of Segovia (Autonomous Community of Castilla-León), included subjects from 14 small and middle-sized towns. Assuming a prevalence of MetS of 20  % according to previous data [16], it was calculated that it would be necessary to recruit from the census a random sample of 2,992 individuals aged 35 to 74 years old (target population of 63,417 inhabitants, 62 % rural). Nevertheless, individuals who agreed to participate were 1,166 (response rate, 39 %), and, from those, only 900 completed the survey. For the final analysis, 13 cases were excluded as blood pressure was not obtained accurately. In summary, 7,115 males and nonpregnant females aged 35 to 74 years old were invited to participate from a targeted population of 412,397 subjects from 21 small and middle-sized towns across Spain, and 3,816 (1,741 males and 2,075 females) were finally included (overall response rate 53.8 %). In both studies, subjects with type 1 diabetes mellitus, heart failure or hepatic insufficiency, surgery in the previous year, abdominal wall hernias, weight loss or gain ≥ 5 kg in the previous six months, or institutionalized were excluded. All subjects were sent a personalized letter signed by the principal investigator and the Regional Public Authorities, explaining the purpose of the study and requesting volunteering for participation. In case of no response, people were again contacted by telephone up to three times. The standard procedures were adapted from the WHO MONICA protocol (WHO, 1990) [17] and approved by the respective ethics committees. All participants were given written information and signed the informed consent. A medical questionnaire was obtained by trained interviewers, requesting from each participant data related to demographic characteristics, including age, sex, education status, socioeconomic status, physical activity, cigarette smoking, alcohol consumption, family history of diabetes and its treatment, hypertension, and other selected chronic diseases.

Anthropometrics measurements were performed using standardized protocols and included weight, height, and waist circumference (in cm). The waist circumference (WC) was measured three times using an anthropometric tape while study participants were standing erect in a relaxed position with both feet together on a flat surface at the smallest horizontal girth between the costal margins and the iliac crests at minimal respiration and averaged for analysis. Body mass index (BMI) was defined as weight (kg) divided by the square of height (m^2^). Blood pressure (BP) was averaged from three attended measures performed in a resting and sitting position by own subjects' primary care physicians, or alternatively trained technicians, after a 10-minute seated rest. A minimum interval of 5 minutes was observed within the three measures, carried out with a random-zero mercury sphygmomanometer with an appropriate sized cuff, and following a standard protocol. Systolic BP and diastolic BP were defined as the points of the appearance and disappearance of Korotkoff sounds, respectively.

Information on pharmacological treatment of hypertension and elevated glucose was based on the participant's reported use of any medication and the transcription and coding of all medication names.

Educational status was estimated as the highest number of completed schooling years [18]. Social class classification was estimated according to the type of job or professional activity as described [18]. Alcohol intake was categorized in the following intervals: no alcohol intake 0 g alcohol/day, 1–14.99 g/day, ≥ 15–29.99 g/day, and ≥ 30 g/day [19, 20]. Smoking was grouped in three categories: current (at least one cigarette per day); never (those who had never smoked); and former (people who quit smoking >1 year ago at the time of the study) [21].

### 2.1. Procedures and Laboratory Studies

Hypertension was diagnosed in those subjects treated with blood pressure medication and/or had a mean systolic BP ≥ 140 mm Hg or alternatively equal or higher of diastolic BP ≥ 90 mm Hg, according to the guidelines of the European Hypertension Society [22]. BP control was defined as < 140/90 mm Hg in nondiabetic subjects and < 140/85 mm Hg in type 2 diabetic subjects [22]. Individuals with a history of hyperlipidemia, hypertension, or diabetes mellitus were deemed to have their respective risk factors, regardless of the biochemical values. Subjects were considered obese if their BMI was ≥ 30 kg/m^2^.

After an overnight period, 20 ml of blood were obtained from an antecubital vein without compression. Plasma glucose concentration was determined twice by a glucose-oxidase method adapted to an Autoanalyzer (Hitachi 704, Boehringer Mannheim, Germany). Total cholesterol, triglycerides, and high-density lipoprotein (HDL-C) cholesterol were determined by enzymatic methods using commercial kits (Boehringer Mannheim, Germany). Low-density lipoprotein (LDL-C) cholesterol was calculated by the Friedewald formula. A 75 g oral glucose tolerance test (OGTT) was performed and interpreted according to the 2003 criteria of the American Diabetes Association [23] after excluding clinically diagnosed diabetic patients. DM was analytically diagnosed when fasting plasma glucose (FPG) was ≥ 7.0 mmol/l (≥ 126 mg/dl) or 2-h glucose ≥ 11.1 mmol/l (≥ 200 mg/dl). Subjects on antidiabetic medications were also considered to have diabetes. In nondiabetic subjects, prediabetes was diagnosed in any of the following cases: IFG was defined as FPG 5.6–6.9 mmol/l (100–125 mg/dl), IGT as 2-h glucose 7.8–11.0 mmol/l (140–199 mg/dl), and IFG/IGT as FPG 5.6–6.9 mmol/l (100–125 mg/dl) and 2-h glucose 7.8–11.0 mmol/l (140–199 mg/dl).

### 2.2. Statistical Methods

Student t-test or analysis of variance ANOVA test were used to compare continuous variables expressed as means ± standard deviation (SD). The level of significance was set at 0.05 for all analyses. Linear regression was used to calculate quantitative variables adjusted for age and sex and their 95 % confidence intervals (CI). Age-standardized rates were based on direct standardization using the Spanish Population Census obtained from the Spanish Statistic Institute (www.ine.es). Otherwise, multivariate logistic regression analyses were performed to evaluate associations of age, body mass index, diabetes, cardiovascular disease, hypercholesterolemia, education level, alcohol, and smoking habits with being hypertensive and with the risk of being blood pressure uncontrolled. Adjusted Odds Ratios (ORs) and their 95 % CI were calculated. All analyses were performed using STATA software (version 11.0; StataCorp, College Station, TX, USA).

## 3. Results

We included 3,816 subjects (Table 1(a)) with no differences in the mean age between sexes, but subjects from rural areas were close to 2 years older (men p=.006, women p<.001). No differences between areas were found in both sexes for BMI, microalbuminuria, number of obesity subjects, known and unknown type 2 DM, known hypertension, and coronary and cerebrovascular diseases. WC was higher in urban versus rural men (um 95.45 versus rm 93.99 cm, p=.003). Diastolic blood pressure was different according to areas for both sexes (um 80.81 versus rm 78.82 mm Hg, p<.001; uw 79.24 versus rw 78.15, p=.035), as well as systolic blood pressure in women (uw 127.97 versus rw 126.05, p=.043). Rural diabetic women were more aware of suffering the disease than their urban counterparts (uw 3.01 versus rw 5.07 %, p=.024). More prediabetic men were found in the rural area (30 versus 24.4 %, p=.041). Known dyslipidemia was more prevalent in the urban area in both sexes (um 61.97 versus rm 52.28 %, p<.001; uw 60.00 versus rw 52.57 %, p=.001). Regarding habits (Table 1(b)), there was a higher alcohol intake in the rural area in both sexes [men: moderate (um 30.36 versus rm 33.59 %) and heavy drinkers (um 24.68 versus rm 30.42 %), p=.001; women: moderate (uw 13.27 versus rw 17.51 %), and heavy drinkers (uw 1.42 versus rw 2.10 %), p=.030], but more current smokers in the urban setting [men: um 43.62 versus rm 39.77 %, p<.001; women: (uw 20.32 versus rw 11.79 %), p<.001]. A higher degree of achieved studies was also found for both sexes in urban areas [men: secondary studies (um 53.51 versus rm 58.12 %) and third degree studies (um 22.11 versus rm 7.36 %), p=.001; women: secondary studies (uw 43.56 versus rw 57.78 %) and third degree studies (uw 16.21 versus rw 10.12 %), p<.001], as well as a higher number of unemployed and lower number of manual workers in both sexes in the urban area (p<.001).

### 3.1. Hypertension (Diagnosed and Undiagnosed) and Blood Pressure Control

The age-standardized prevalence of hypertension was 25.45 % (CI 95 %: 23.76 – 27.14). According to sex, the age-standardized prevalence of hypertension was 21.39 % (CI 95 %: 19.13 – 23.65) in men and 29.10 % (CI 95 %: 26.59 – 31.62) in women. The prevalence of hypertension (Table 2(a)) increased with age (13.66, 25.92, 28.74 % / p<.001 in men and 12.64, 35.18, 45.81 % / p<.001 in women, aged 31-45, 46-60, and 61-77 years old, respectively) with no differences between areas in the age's groups considered. Regarding the prevalence of undiagnosed hypertension (Table 2(b)), we found a 16.68 % (CI 95 %: 14.98 – 18.39) of age-standardized prevalence. According to sex, the age-standardized prevalence of undiagnosed hypertension was 17.29 % (CI 95 %: 14.91 – 19.68) in men and 16.38 % (CI 95 %: 13.87 – 18.88) in women. The prevalence of undiagnosed hypertension also increased with age (11.39, 17.50, and 27.00 %/ p<.001 in men and 6,02, 17.88, and 29.41 %/ p<.001 in women aged 31-45, 46-60, and 61-77 years old, respectively). Interestingly, there was a 5 % higher prevalence of undiagnosed hypertension in urban versus rural women aged 46-60 years old (uw 19.85 versus rw 14.18 %, p=.018).

**Figure 1:**
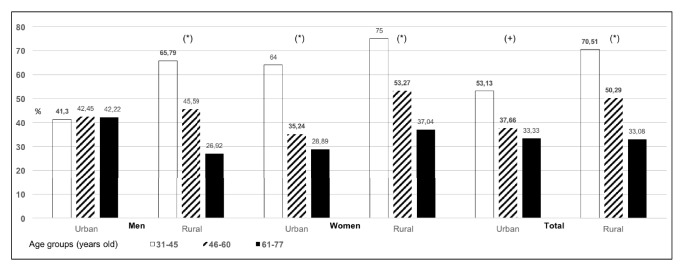
**Prevalence of controlled blood pressure in hypertensive subjects according to the age group and living area**. (*∗*) Overall age categories comparison p<.001 (+). Overall age categories comparison p=.004.  Comparatives:** 31 to 45 year-old urban versus rural men** (**41.30 versus 65.79 %, p=.025**), 31 to 45 year-old urban versus rural women (64.00 versus 75.00 %, p=.263),** 31 to 45 year-old urban versus rural subjects** (**53.13 versus 70.51 %, p=.019**), 46 to 60 year-old urban versus rural men (42.45 versus 45.59 %, p=.684),** 46 to 60 year-old urban versus rural women (35.24 versus 53.27 %, p=.002), 46 to 60 year-old urban versus rural subjects** (**37.66 versus 50.29 %, p=.002**), 61 to 77 year-old urban versus rural men (42.22 versus 26.92 %, p=.113), 61 to 77 year-old urban versus rural women (28.89 versus 37.04 %, p=.257), 61 to 77 year-old urban versus rural subjects (33.33 versus 33.08 %, p=.965). Statistically significant values are highlighted in bold letters.

The prevalence of BP control (Figure 1) decreased significantly with age in rural but not urban men [um 41.30, 42.45, and 42.22 % (p >.05), rm 65.79, 45.59, and 26.92 % (p<.001) aged 31-45, 46-60, and 61-77 years old, respectively] and for urban and rural women [uw 64.00, 35.24, and 28.89 % (p<.001); rw 75.00, 53.27, and 37.04 % (p<.001) aged 31-45, 46-60, and 61-77 years old, respectively]. The BP control was higher in younger (aged 31 to 45 old) hypertensive rural men as compared to urban (uw 65.79 versus rw 41.30 %, p=.025). Similarly occurred with medium aged (46 to 60 years old) rural women (uw 35.24 versus rw 53.27 %, p=.002). No differences were found regarding the BP control in urban versus rural area populations at other age's categories.

Multivariate-adjusted logistic regression analyses showed that the probability of being hypertensive is higher in older and obese men and women, women with prediabetes or history of cardiovascular disease, nonsmoker women, and hypercholesteraemic men and women (Table 3). Women with secondary studies were less frequently diagnosed with hypertension [OR 0.486 (0.310-0.761), p <.001], oppositely to nonsmoker women [OR 3.703 (1.866-7.349), p<.001]. Uncontrolled blood pressure (Table 4) was most frequent in men with diabetes [OR 6.460 (1.260-33.125), p=.025] or nonsmokers [OR 3.126 (1.012-9.655), p=.048], while women living in rural areas [OR 0.501 (0.258-0.970), p=.040] and men with secondary or tertiary education levels [OR 0.245 (0.092-0.654), p=.005, and OR 0.156 (0.044–0.549), p=.004, respectively] were more prone to be controlled. Women with secondary or tertiary education levels had a trend towards better BP control [OR 0.467 (0.211–1.038), p=.060, and OR 0.337 (0.108–1.046), p=.060].

### 3.2. Pharmacological Therapy

Most of pharmacologically treated subjects were on monotherapy (data shown in Supplementary Table 1). Most frequent used drugs were diuretics and angiotensin converting enzyme inhibitors (32.1 and 30.3 % of subjects, respectively). The most frequent combined therapy was a diuretic with an inhibitor of the angiotensin converting enzyme (30 %).

## 4. Discussion

In this adult population from Spain, the prevalence of known hypertension is higher in women than men (29.23 versus 21.90 %) without differences between areas. In contrast, in a recent nationwide population-based study from Spain [24], the prevalence of known and unknown hypertension in subjects of similar mean age was significantly high (42.6 and 37.4 %, respectively). Nevertheless, that study was designed to report on the prevalence of diabetes mellitus type 2 (DM2) and had a higher proportion of DM2 subjects than our study. Another important study [25] included prevalence data on hypertension from 6 European countries. The hypertension prevalence in Spain was 49.0 % in men and 44.6 % in women (age range 35 to 65 years old), that together with other European countries represented a 60 % higher prevalence than the reported in United States and Canada. Other representative study of the Spanish Population [26], the* ENRICA* study, found a prevalence of hypertension of 33.3 %, more in accordance with our findings. Interesting from this study was that only 59.4 % of the subjects were aware of their condition and only 48.5 % of them were blood pressure (BP) controlled. Authors reported that education level was influencing BP control, in correlation to our finding that men with secondary or tertiary education levels had a lower probability of being BP uncontrolled.

In the northeast of Spain, prevalence of hypertension was higher to the herein reported, with one out of three subjects diagnosed with hypertension and, interestingly, near 1 of 2 subjects suffering from unknown hypertension [27]. There was a significant correlation with alcohol intake, obesity, and family history of hypertension or cardiovascular disease, and no correlation with professional level, education, or hypertension in the spouse. Probably, the main limitation of this study was the number of participating subjects (n = 670), too low for a comprehensive study of hypertension associated variables. On the other hand, a larger study with near 3,000 subjects in the northwest of Spain [10] found that prevalence of hypertension was higher in subjects with low educational level in which close association was observed with cardiovascular diseases. The authors reported that one up to four subjects had a diagnosis of hypertension, in accordance with our study results, and as in the previously mentioned ENRICA study [26], only 1 of 2 subjects was aware of its hypertensive status. Another population-based study in subjects aged ≥ 60 years old found that BP control was related to living in rural areas, as we have found in rural women, being uncoupled or doing moderate physical activity in men, as well as drinking moderate alcohol in women [11]. Relevant of this study is that it is one of the few studies in Spain that clearly defines and addresses the living area, a factor that in our opinion should be considered in the study of hypertension prevalence and incidence, as a consequence of the different ways of live [10–12, 16]. In fact, the type of diet and the physical activity are two of the seven defined factors involving cardiovascular health according to the* American Heart Association* [28]. On the other hand, it has been reported that underserved rural areas had higher rates of hypertension diagnosis as well as other cardiovascular risk factors, but also high rates of uncontrolled BP [4]. For this reason, achieving a similar degree of BP control in rural versus urban areas could be an indicator of a better and more widespread healthcare system. Thus, it is noticeable that we have found a better BP control in rural women but not men after adjusting for multiple confounders. The reason for this finding is not clear and could be a consequence of differences in lifestyle across areas in women, such as diet or exercise, rather than differences in the access to the healthcare system.

More recently, a large study in Italy, including ten thousand subjects with a mean age of 56 years old, confirmed a high prevalence of hypertension (between 55.4 and 59.0 %) in the real life setting for the period of 2004 to 2014, with a slight tendency over a better BP control over the 10-year period (from 50 to 57.6 %). In contrast, we found a lower proportion of patients with an adequate BP control, higher in women than men and lowering with increasing age. Moreover, less than one in three patients achieves optimal BP control after the age of 60 in our study. Also in Italy, in a recent study with near 10,000 outpatients from 1,666 primary care physicians' consultations, Tocci et al. [29] found 72.5 % of hypertension diagnosed subjects. The main reported result was that less than a third of hypertensive subjects (30 %) achieved the recommended BP target levels in Italy, results in accordance with our study and other studies in Spain [30].

Our findings in the prescribed pharmacologic therapy differ with other studies, as we have found a significant high prescription of diuretics in the monotherapy group. The reason could be related to the years of recruitment of our study, as diuretics were highly prescribed as a first line therapy in the 80s and 90s of the past century and our subjects were recruited at the end of the 90s and 2001-2002. In fact, diuretics occupied the fourth hypertension treatment position in the PRESCAP study in 2002 (prescribed in 10.6 % of men and 18.8 % of women), while eight years later, in the PRESCAP 2010 study, a downward trend in the prescription of diuretics as monotherapy was highlighted (7.3 % in men and 17.2 % in women) [31]. Angiotensin converting enzyme drugs were the second more prescribed pharmacologic group class after diuretics in our population, although these drugs are already the first therapeutic prescribed class in other studies [24, 29].

## 5. Study Limitations

Causal inferences from our data are not possible because of the cross-sectional design. Otherwise, the reduction in the initial sample size could have conducted to a nonrepresentative population study. Due to this fact, we compared our cohorts (age, sex distribution, and area frequencies of subjects finally included in the study) to the Census of the National Institute of Statistics of Spain (www.ine.es) for the same years and found that they were nearly identical. We did not assess physical exercise and nutrient intake story through dietary standardized questionnaire, missing important information as, for example, the degree of adherence to Mediterranean diet, that is associated with a higher prevalence of hypertension and related factors. Other potential biases are as follows: first, estimated prevalence of hypertension might be too high because healthy population could have declined to participate; second, alcohol consumption was self-reported so it could be underestimated; and third, information on ambulatory BP or at least a second day BP measurement was not available; thus we cannot provide data on proportions of patients achieving sustained BP control.

## 6. Conclusions

The prevalence of diagnosed hypertension in a Caucasian population of Spain was higher in women and showed no differences according to the living area (urban versus rural) in both sexes. Women living in rural areas and men with secondary or tertiary education levels have a lower probability of being blood pressure uncontrolled. Urban young men (31-45 years old) and medium aged women (46-60 years old) are less blood pressure controlled than their rural counterparts.

## Figures and Tables

**Table:** (a) Clinical characteristics of the study population according to sex and living area.

	**Men**				**Women**			
	**Urban**	**Rural**	**p**	**Total**	**Urban**	**Rural**	**P**	**Total**

**N (%)**	952 (54.68)	789 (45.32)		1,741 (45.62)	1,265 (60.96)	810 (39.04)		2,075 (54.38)

**Age (years),** mean ± SD	**49.96 ± 9.51**	**51.27 ± 10.50**	**0.006**	50.55 ± 9.99	**49.97 ± 9.33**	**51.92 ± 10.54**	**< 0.001**	50.73 ± 9.87

**Age ranges (years)**								

31-45 n (%)	**371 (38.97)**	**288 (36.50)**	**0.001**	659 (37.85)	**470 (37.15)**	**279 (34.44)**	**< 0.001**	749 (36.10)
46-60 n (%)	**420 (44.12)**	**309 (39.16)**		729 (41.87)	**604 (47.75)**	**329 (40.62)**		933 (44.96)
61-77 n (%)	**161 (16.91)**	**192 (24.33)**		353 (20.28)	**191 (15.10)**	**202 (24.94)**		393 (18.94)

**BMI **(kg/m2),	27.65 ± 3.58	27.45 ± 3.62	0.230	27.56 ± 3.60	28.04 ± 4.86	27.96 ± 4.99	0.720	28.01 ± 4.91
mean ± SD								

**Waist circumference **(cm), mean ± SD	**95.45 ± 10.12**	**93.99 ± 9.79**	**0.003**	94.79 ± 10.00	85.85 ± 11.50	85.87 ± 10.83	0.965	85.86 ± 10.83

**SBP **(mm Hg),	127.89 ± 18.61	126.39 ± 8.33	0.093	127.21 ± 18.50	**127.97 ± 21.63**	**126.05 ± 20.17**	**0.043**	127.22 ± 21.09
mean ± SD								

**DBP **(mm Hg), mean ± SD	**80.61 ± 11.20**	**78.82 ± 10.88**	**< 0.001**	79.81 ± 11.09	**79.24 ± 11.64**	**78.15 ± 11.12**	**0.035**	78.82 ± 11.45

**Microalbuminuria **(mg/l), median (p25-p75).	5.9 (3.9 – 9.0)	5.3 (3.5 – 10.2)	0.146	5.55 (3.5 – 9.6)	4.0 (2.5 – 7.3)	4.2 (2.5 – 7.9)	0.804	4.2 (2.5 – 7.7)

**Obesity **(%)	23.63	22.59	0.609	23.16	30.70	32.13	0.494	31.26
(BMI ≥ 30 kg/m2)								

**Diabetes Mellitus **(%) (unknown + known)	8.93	8.08	0.546	8.54	6.72	7.48	0.526	7.02

**Diabetes Mellitus **(%) (known)	5.08	4.93	0.744	5.02	**3.01**	**5.07**	**0.024**	3.82
**Diabetes Mellitus **(%) (unknown)	3.84	3.15		3.53	**3.70**	**2.40**		3.19

**Prediabetes **(%)	**24.41**	**30.00**	**0.041**	26.93	20.59	22.43	0.465	21.31
(IFG + IGT)								

**Coronary disease **(%)	3.36	2.53	0.311	2.98	1.19	1.11	0.868	1.16

**Cerebrovascular disease **(%)	1.47	1.52	0.934	1.49	0.71	0.86	0.704	0.77

**Peripheral artery disease** (%)	0.10	0.38	0.232	0.23	**-* *--**	**-* *--**	**-* *--**	**-* *-- (** **∗** **)**

**Known hypertension **(%)	21.88	21.92	0.986	21.90	28.73	30.01	0.540	29.23

**Known dyslipidemia **(%)	**61.97**	**52.28**	**< 0.001**	57.57	**60.00**	**52.57**	**0.001**	57.08

(*∗*) No cases were reported. IFG: Impaired Fasting Glucose. IGT: Impaired Glucose Tolerance. Statistically significant values (p< 0.05) are highlighted in bold letters.

**Table:** (b) Habits, education, and socioeconomic status of the study population according to sex and living area.

	**Men**				**Women**			

	**Urban**	**Rural**	**p**	**Total**	**Urban**	**Rural**	**p**	**Total**

**Alcohol intake **(%)								

Never	**19.96**	**17.74**	**0.001**	18.95	**56.16**	**53.88**	**0.030**	55.27
Occasionally	**25.00**	**18.25**		21.94	**29.15**	**26.51**		28.12
Moderate	**30.36**	**33.59**		31.82	**13.27**	**17.51**		14.93
Heavy drinker	**24.68**	**30.42**		27.28	**1.42**	**2.10**		1.69

**Smoking habit **(%)								

Smoker	**43.62**	**39.77**	**< 0.001**	41.87	**20.32**	**11.79**	**< 0.001**	16.97
Non smoker	**19.07**	**28.16**		23.21	**67.79**	**79.16**		72.26
Formersmoker	**37.30**	**32.07**		34.92	**11.89**	**9.06**		10.78

**Education level **(%)								

Illiterate	**21.23**	**32.74**	**< 0.001**	25.93	**31.55**	**27.41**	**< 0.001**	30.02
Primary studies	**3.16**	**1.78**		2.59	** 8.68**	**4.69**		7.21
Secondary studies	**53.51**	**58.12**		55.39	**43.56**	**57.78**		48.81
Third degree studies	**22.11**	**7.36**		16.08	**16.21**	**10.12**		13.96

**Socioeconomic status **(%)								

Student	**0.11**	**-* *-- (** **∗** **) **	**< 0.001**	0.06	**-* *-- (** **∗** **)**	**0.13**	**< 0.001**	0.06
Retired	**23.31**	**25.16**		24.17	**41.17**	**38.58**		40.08
Unemployed	**10.29**	**4.08**		7.41	**10.65**	**4.86**		8.20
Manual worker	**36.46**	**43.74**		39.84	**28.69**	**36.09**		31.82
Other jobs	**29.83**	**27.01**		28.52	**19.48**	**20.34**		19.84

(*∗*): no cases were reported. Statistically significant values (p< 0.05) are highlighted in bold letters.

**Table:** (a) Prevalence of diagnosed hypertension by age groups and living area in the sample.

	**Age groups (years)**											
	**31 - 45**				**46 – 60**				**61-77**			

%	**Urban**	**Rural**	**p**	**Total **(95 % CI)	**Urban**	**Rural**	**p**	**Total **(95 % CI)	**Urban**	**Rural**	**P**	**Total **(95 % CI)

**Men **	13.26	14.18	0.741	13.66 (11.04 – 16.63) [*∗*]	26.60	24.91	0.620	25.92 (22.67 – 29.37) [*∗*]	29.11	28.42	0.887	28.74 (23.99 – 33.86) [*∗*]

**Women**	11.11	15.27	0.108	12.64 (10.29 – 15.31) [+]	35.59	34.39	0.719	35.18 (32.06 – 38.39) [+]	49.20	42.56	0.193	45.81(40.73 – 50.95) [+]

**Total**	12.05	14.72	0.158	13.11 (11.34 – 15.05)	31.93	29.95	0.411	31.19 (28.92 – 33.53)	40.00	35.71	0.235	37.76 (34.21 – 41.41)

[*∗*] p < 0.001 for the comparison of the three age categories in men. [+] p < 0.001 for the comparison of the three age categories in women.

**Table:** (b) Prevalence of undiagnosed hypertension by age groups and living area in the sample.

	**Age groups (years)**											
	**31 - 45**				**46 – 60**				**61-77**			

**%**	**Urban**	**Rural**	**p**	**Total **(95 % CI)	**Urban**	**Rural**	**p**	**Total **(95 % CI)	**Urban**	**Rural**	**p**	**Total **(95 % CI)

**Men**	11.37	11.40	0.991	11.39 (8.95 – 14.38) [*∗*]	19.87	14.08	0.093	17.50 (14.42 – 21.06) [*∗*]	29.36	25.00	0.451	27.00 (21.75 – 32.99) [*∗*]

**Women**	6.09	5.88	0.917	6.02 (4.40 – 8.18) [+]	19.84	14.29	0.097	17.88 (14.97 – 21.22) [+]	25.53	32.73	0.261	29.41 (23.59 – 36.00) [+]

**Total**	8.37	8.69	0.851	8.49 (7.01 – 10.25)	**19.85**	**14.18**	**0.018**	17.70 (15.54 – 20.09)	27.59	28.57	0.819	28.12 (24.12 – 32.49)

(*∗*) p < 0.001 for the comparison of the three age categories in men. (+) p < 0.001 for the comparison of the three age categories in women. The statistical significance is highlighted in bold letters.

**Table 3:** Multiple logistic regression analysis of subjects' probability of being hypertensive after adjusting for age, body mass index, diabetes, cardiovascular disease, hypercholesterolemia, education level, alcohol, and smoking habits.

	**Men**		**Women**	
	**OR (95 % CI)**	**p**	**OR (95 % CI)**	**p**

**Living area**				

Urban area	1		1	

Rural area	0.905 (0.614 – 1.334)	0.614	1.017 (0.703 – 1.472)	0.927

**Age categories (years)**				

31 – 45	1		1	

46 - 60	**1.863 (**1.212 – 2.862)	** 0.005**	**1.679** (1.078 – 2.616)	**0.022**

61 - 77	**2.717** (1.473 – 5.010)	** 0.001**	1.635 (0.895 – 2.986)	0.110

**BMI (kg/m2)**				

< 30	1		1	

≥ 30	**1.943** (1.281 – 2.949)	** 0.002**	**3.219** (2.228 – 4.650)	**< 0.001**

**Diabetes Mellitus**				

No	1		1	

Prediabetes	1.041 (0.685 – 1.582)	0.852	**1.823 **(1.228 – 2.706)	**0.003**

Diabetes	**0.799** (0.400 – 1.598)	**< 0.001**	1.040 (0.529 – 2.043)	0.910

**Cardiovascular disease**				

No	1		1	

Yes	2.129 (0.978 – 4.638)	0.057	**4.029** (1.369 – 11.862)	**0.008**

**Hypercholesterolemia**				

No	1		1	

Yes	**1.670** (1.148 – 2.525)	**0.008**	**1.651 **(1.151 – 2.369)	**0.007**

**Education level**				

None	1		1	

Primary	1.084 (0.311 – 3.780)	0.863	1.076 (0.555 – 2.085)	0.828

Secondary	0.984 (0.601 – 1.611)	0.950	**0.486 **(0.310 – 0.761)	**0.002**

Tertiary	0.798 (0.419 – 1.519)	0.550	0.802 (0.428 – 1.505)	0.493

**Alcohol intake**				

No	1		1	

Occasionally	0.881 (0.491 – 1.579)	0.669	0.855 (0.555 – 1.315)	0.475

Low-Moderate	0.840 (0.486 – 1.453)	0.533	0.982 (0.595 – 1.622)	0.945

Heavy	1.143 (0.661 – 1.976)	0.631	0.836 (0.193 – 3.627)	0.810

**Smoking habit**				

Yes	1		1	

No	0.795 (0.471– 1.342)	0.391	**3.703** (1.866 – 7.349)	**< 0.001**

Former	0.961 (0.632 – 1.464)	0.857	2.126 (0.929 – 4.868)	0.074

Statistically significant values (p< 0.05) are highlighted in bold letters.

**Table 4:** Multiple logistic regression analysis of hypertensive treated subjects of being blood pressure uncontrolled after adjusting for age, body mass index, diabetes, cardiovascular disease, hypercholesterolemia, education level, alcohol, and smoking habits.

	**Men**		**Women**	
	**OR (95 % CI)**	**p**	**OR (95 % CI)**	**p**

**Living area**				

Urban area	1		1	

Rural area	0.819 (0.380 – 1.765)	0.610	**0.501 **(0.258 – 0.970)	**0.040**

**Age categories (years)**				

31 – 45	1		1	

46 - 60	0.933 (0.392 – 2.220)	0.876	1.595 (0.650 – 3.917)	0.308

61 - 77	1.187 (0.387 – 3.642)	0.765	2.045 (0.651 – 6.424)	0.220

**BMI (kg/m2)**				

< 30	1		1	

≥ 30	0.757 (0.342 – 1.675)	0.492	1.403 (0.752 – 2.617)	0.287

**Diabetes Mellitus**				

No	1		1	

Prediabetes	0.769 (0.345 – 1.714)	0.520	1.898 (0.990 – 3.642)	0.054

Diabetes	**6.460** (1.260 – 33.125)	**0.025**	1.799 (0.554 – 5.839)	0.329

**Cardiovascular disease**				

No	1		1	

Yes	1.376 (0.360 – 5.270)	0.641	0.967 (0.241 – 3.881)	0.963

**Hypercholesterolemia**				

No	1		1	

Yes	0.819 (0.350 – 1.918)	0.646	0.596 (0.305 – 1.166)	0.131

**Education level**				

None	1		1	

Primary	0.855 (0.685 – 10.666)	0.903	1.180 (0.425 – 3.277)	0.751

Secondary	**0.245 **(0.092 – 0.654)	**0.005**	0.467 (0.211 – 1.038)	0.062

Tertiary	**0.156 **(0.044 – 0.549)	**0.004**	0.337 (0.108 – 1.046)	0.060

**Alcohol intake**				

No	1		1	

Occasionally	1.181 (0.356 – 3.917)	0.785	0.560 (0.250 – 1.255)	0.159

Low-Moderate	1.760 (0.562 – 5.512)	0.332	0.531 (0.221 – 1.272)	0.155

Heavy	2.258 (0.720 – 7.08)	0.163	1.082 (0.069 – 16.989)	0.955

**Smoking habit**				

Yes	1		1	

No	**3.126 **(1.012 – 9.655)	**0.048**	1.237 (0.282 – 5.430)	0.778

Former	1.828 (0.789 – 4.234)	0.159	0.914 (0.153 – 5.473)	0.922

Statistically significant values (p< 0.05) are highlighted in bold letters.

## Data Availability

The datasets generated and analyzed during the current study are not publicly available as they contain information that could compromise research participant privacy, but they are available from the corresponding author on reasonable request.

## Supplementary Materials

Supplementary Materials Supplementary Table 1. Pharmacologic hypertensive drug prescriptions in the treated subjects.
