# Supplementary material for: Prevalence, Treatment, and Associated Factors of Hypertension in Spain: A Comparative Study between Populations
Source: Int J Hypertens. 2018 Aug 14;2018:4851512. doi: 10.1155/2018/4851512 (PMC6112205; doi:10.1155/2018/4851512)
Supplement: Supplementary Materials — Supplementary Table 1. Pharmacologic hypertensive drug prescriptions in the treated subjects. [file 4851512.f1.docx]

**Supplementary Table 1. Pharmacologic hypertensive drug prescriptions in the treated subjects.**

| **Blood pressure lowering treatment** | **n** | **%** |
| --- | --- | --- |
| **MONOTHERAPY** | 109 | 68.55 |
| ACEIs | 33 | 30.3 |
| ARBs | 8 | 7.3 |
| Diuretics | 35 | 32.1 |
| ß-Blockers | 11 | 10.1 |
| CCBs | 16 | 14.7 |
| **Other treatments** | 6 | 5.5 |
| **COMBINED THERAPY** | 50 | 31.45 |
| Diuretics + ACEIs | 15 | 30 |
| Diuretics + ARBs | 6 | 12 |
| Diuretics + CCBs | 5 | 10 |
| ß-Blockers + CCBs | 1 | 2 |
| ß-Blockers + ACEIs | 1 | 2 |
| ß-Blockers + ARB | 1 | 2 |
| ß-Blockers + diuretics | 5 | 10 |
| ACEIs + CCBs | 2 | 4 |
| ARBs + CCBs | 1 | 2 |
| **Other combinations of 2 drugs** | 1 | 2 |
| **Diuretics + ACEIs + CCBs** | 3 | 6 |
| **Other combination of 3 drugs** | 9 | 18 |

Abbreviations: ACEI: angiotensin-converting enzyme inhibitor; ARB: angiotensin II receptor blocker; CCB: calcium channel blocker.
